# Laparoscopic Surgery in COVID-19 Era—Safety and Ethical Issues

**DOI:** 10.3390/diagnostics10090673

**Published:** 2020-09-04

**Authors:** Dragos Serban, Catalin Gabriel Smarandache, Corneliu Tudor, Lucian Nicolae Duta, Ana Maria Dascalu, Cătălin Aliuș

**Affiliations:** 1 Faculty of Medicine, “Carol Davila” University of Medicine and Pharmacy Bucharest, 030167 Bucharest, Romania; gabriel.smarandache@umfcd.ro (C.G.S.); ana.dascalu@umfcd.ro (A.M.D.); 2 IVth Department of Surgery, Emergency University Hospital Bucharest, 050098 Bucharest, Romania; lulu.tudor@gmail.com (C.T.); Lucian.duta@gmail.com (L.N.D.); alius.catalin@gmail.com (C.A.)

**Keywords:** laparoscopy, aerosolization, COVID-19, filtration, surgical plume

## Abstract

(1) Background: The paper aims to review the available evidence regarding the health risk of the aerosolization induced by laparoscopy induced and impact of the COVID-19 pandemic upon minimally invasive surgery. (2) Materials and methods: A systematic review of the literature was performed on PubMed, Medline and Scopus until 10 July. (3) Results: Chemicals, carcinogens and biologically active materials, such as bacteria and viruses, have been isolated in surgical smoke. However, the only evidence of viral transmission through surgical smoke to medical staff is post-laser ablation of HPV-positive genital warts. The reports of SARS-CoV-2 infected patients who underwent laparoscopic surgery revealed the presence of the virus, when tested, in digestive wall and stools in 50% of cases but not in bile or peritoneal fluid. All surgeries did not result in contamination of the personnel, when protective measures were applied, including personal protective equipment (PPE) and filtration of the pneumoperitoneum. There are no comparative studies between classical and laparoscopic surgery. (4) Conclusions: Previously published data showed there is a possible infectious and toxic risk related to surgical smoke but not particularly proven for SARS-CoV-2. Implementing standardized filtration systems for smoke evacuation during laparoscopy, although increases costs, is necessary to increase the safety and it will probably remain a routine also in the future.

## 1. Introduction

COVID-19 pandemic has a deep impact on the social and economic life worldwide and is considered the most severe sanitary crisis since the Spanish flu, a hundred years ago. The etiologic agent, called SARS-CoV-2, is an RNA virus in the Coronavirus family, generally responsible for benign respiratory infections, except for the causative agents of Middle East respiratory syndrome (MERS) in 2012 and severe acute respiratory syndrome (SARS) during 2002–2003 outbreaks [1]. With an estimated fatality rate of 0.5–1% [2] of the total number of infected people and 5% of those diagnosed [3], SARS-CoV-2 infection remains a global threat, months after the onset of the pandemic, due to the unpredictability of the clinical course in subjects in apparent good health, the extremely high contagiousness and the absence of specific antiviral treatment. Pulmonary decompensation may occur suddenly, after days of asymptomatic or oligosymptomatic evolution of the disease, with O_2_ desaturation, requiring ventilatory support. Although comorbidities and advanced age are factors statistically associated with increased mortality, the existence of severe forms in young adults and among medical staff has increased the psychological pressure of those treating patients suspected or infected with SARS-CoV-2.

The ways of intra-community transmission of the infection are via droplets, in the area of 1–2 m from the infected person, with possible oronasal or conjunctival entrance gates, by contact with infected surfaces and by inhalation of aerosols with viral load. Aerosols are smaller particles, under 5 μm, which can travel by air currents and can be inoculated at the entrance gates (nose, eyes, mouth). Due to their small size, they can reach the broncho-alveolar level directly, along with the inspired air [1].

Since the time of SARS-CoV, in 2003, there has been convincing evidence that aerosol-generating procedures (AGP) could potentially result in a wider human-to-human coronavirus transmission radius [4]. Bioaerosols range in size from 0.3 to 100 μm and particles up to 5 μm remain airborne and can travel distances of more than 100 m, which may be a transmission path for SARS-CoV-2. Based on these considerations, as well as taking into account that the SARS-CoV-2 virus was also identified in conjunctival secretions, blood, feces, digestive tract mucosa or cerebrospinal fluid [5], the general recommendations of all medical and surgical societies were to avoid all procedures generating aerosols, including laparoscopic surgery, in patients with uncertain or positive COVID-19 status [6,7,8,9,10,11].

The paper aims to review the available evidence regarding the health risk of the aerosolization induced by laparoscopy induced and impact of the COVID-19 pandemic upon minimally invasive surgery.

## 2. Materials and Methods

A systematic review of the literature was performed on PubMed, Medline and Scopus until 10 July. The following terms were searched, individually or in combination—“pandemic”, “coronavirus”, “COVID-19” and “laparoscopy” or “laparoscopic”. For potentially relevant records, the full-text articles were obtained and the inclusion and exclusion criteria were applied. Original articles, reviews and guidelines in the English language, reporting clinical data and practical indications on operative and perioperative laparoscopic management during COVID-19 pandemics were included. Given the limited available data and the rapid evolution of the pandemic, we included the published articles irrespective of their methodological level and their development process. Editorials, commentaries, duplicated and non-English articles were excluded. The articles regarding the minimally invasive approach in urology and gynecology were also excluded. An additional search was also performed on PubMed/Medline and Scopus, by the words: “laparoscopic smoke”, “composition” and “viral” in order to assess the previous information published about the health risk of laparoscopic smoke and the possibility of viral transmission to the OR personnel during laparoscopic surgery.

## 3. Results

The initial search returned 163 papers published, between March and July 2020 regarding the safety of laparoscopic surgery in COVID-19 suspects and infected patients. Additional search found 79 papers regarding laparoscopic smoke composition and the possible health risk, published before 2020. After the exclusion and inclusion criteria were applied, a total of 43 papers were analyzed. Out of these, 8 were reviews, 7 case/case series reports, 11 recommendations and guidelines and 17 were original articles.

**Figure 1:**
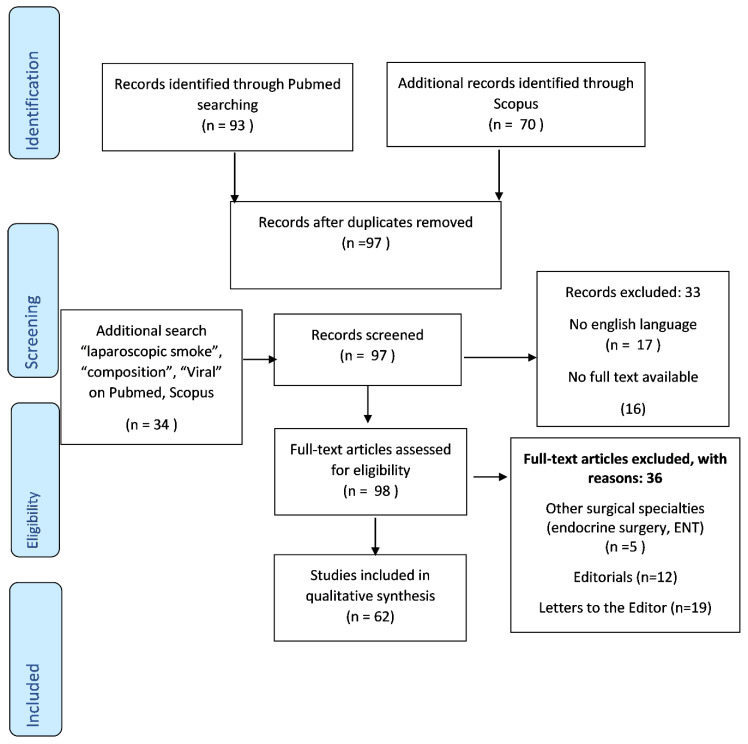
PRISMA (Preferred Reporting Items for Systematic Reviews and Meta-analyses) flow diagram.

The data regarding the possibility of infection by laparoscopic aerosolization of the virus is scarce. Most of the decision-making and guideline development on this topic are based on the limited available data and information inferred from other viruses and similar epidemics and previous researches upon the health risk related to surgical smoke and aerosolization during releasing the pneumoperitoneum. Although there is no direct evidence of transmitting infection via laparoscopy, the SARS-CoV-2 pandemic has caused surgeons the world over to re-evaluate their approach to surgical procedures given concerns over the risk of aerosolization of viral particles and exposure of operating room staff to infection (Figure 1).

### 3.1. Laparoscopy and Aerosolization

The SARS-CoV-2 pandemic brings to attention a neglected subject in previous years, namely that of health risks related to exposure to plume and aerosolization during laparoscopy. Laparoscopic procedures have a theoretical risk of generating aerosols particularly during maintenance and evacuation of a pneumoperitoneum, CO_2_ leakage at the level of trocar orifices or changing instruments and while using energy devices due to smoke generation. Only a few reports in the literature related to the possible risk to the surgical team of inhalation of viruses from patients during a laparoscopy. In 1996, Des Coteaux et al. [12] demonstrated the presence of breathable aerosols and cell-size fragments in the cautery smoke produced during laparoscopic procedures. The discharged product contains 95% water vapor and 5% particles, of very small dimensions, which can pass through the usual surgical mask or can be inoculated at the level of the ocular conjunctiva. The size of the aerosolized particles depends on the type of energy used [12,13,14] (Table 1).

Superior protection consisting of a respirator mask, FFP2 or higher, wrap-around goggles and air filtration devices was discussed even before the Covid-19 pandemic but implemented only partially and non-unitarily [13,15]. The reduction of the pneumoperitoneum insufflation pressure, the reduction of the power and the duration in case of using energy devices are associated with the decrease of the aerosolized particle concentration.

### 3.2. Health Risks Related to Surgical Smoke

Chemicals, carcinogens and biologically active materials, such as bacteria and viruses, have been isolated in surgical smoke. Previous studies show that chronic exposure can cause respiratory problems (cough, laryngitis, chronic bronchitis), eye irritation, headaches and even carcinogenic potential. The main substances identified were—aromatic hydrocarbons (benzene, toluene, ethylbenzene), aldehydes (formaldehyde, benzyl aldehyde, furfural, acrylaldehyde), nitriles, with a possible generation of hydrocyanic acid, furans [14,15,16,17]. Studies have been performed in laboratories showing various pulmonary changes in rats when exposed to smoke plumes [18]. It has been shown previously that 1 g of tissue would create a smoke plume with a mutagenic effect equivalent to smoking 6 unfiltered cigarettes [19,20].

If there is a consensus on the toxic risk of long-term exposure to these harmful substances, we cannot say the same about the risk of infection. Some authors identify viable bacteria in surgical smoke [21]. On the contrary, analysis of the theoretical risk that pneumoperitoneum gas could carry bacteria in aerosol form and spread infection throughout the peritoneal cavity during laparoscopy for infective conditions such as appendicitis was not confirmed in another study, as the pneumoperitoneum gas collected at the end of the procedure did not show any bacterial contamination [22].

Engelhardt et al. [23] demonstrates the aerosolization of blood droplets when pneumoperitoneum is evacuated through the trocar orifices, which can contaminate the operative team at the conjunctival level or by airway. It is estimated that there are cases of conjunctival HIV inoculation and that if the risk of stinging with a surgical needle is 0.5%, the risk of contamination by aerosolization of viral particles in exposed mucous membranes would be 0.1% [23,24].

Aerosolization of blood-borne viruses like hepatitis B virus, HIV and HPV has been previously detected in surgical smoke during laparoscopy [24,25,26,27,28,29,30,31,32,33,34]. However, the only evidence of viral transmission through surgical smoke to medical staff reported in the literature was post-laser ablation of HPV-positive vaginal warts. In 3 cases, gynecologists developed laryngeal papilloma and 2 cases of HPV-16 subtype positive tonsillar squamous cell carcinoma [30,32,34]. It has long been known that live viruses can be isolated from the CO_2_ laser plume, specifically, papilloma and papova virus and that the viral transmission through the respiration of the corneal excimer laser plume is possible and devices to collect and evacuate the plume where created to increased personnel safety [35]. Other authors consider that although RNA presence was demonstrated in the surgical smoke, the previous clinical experience did not show and increased risk for the surgical team [36,37,38]. On the contrary, laparoscopy is considered safer than open surgery due to limited contact with infected blood. The conclusion cannot be applied for SARS-CoV-2, due to different transmission mechanisms of infection. The previously studied viruses are not respiratory viruses. Therefore, potential risk of aerosol exposure must also be considered for SARS-CoV-2. Virus, if present in these particles (<5 μm), can be inhaled and may cause infection (Table 2).

### 3.3. Viral Transmission in Open Versus Laparoscopic Surgery

The previously published studies are on a limited number of cases and the methodology used differs significantly. The conclusion is that viral particles from the tissue treated by laser, electrocautery or ultrasound are present in surgical smoke, along with a multitude of toxic compounds. There are no comparative studies between classical and laparoscopic surgery. On the one hand, minimally invasive surgery has an additional potential to aerosolize when evacuating the pneumoperitoneum or near the trocar holes but on the other hand, if high efficacy filters are used, surgical smoke may be easier to manage than in case of using the same energy devices in open surgery [35,36,37,38,39,40,41,42]. In the current COVID-19 pandemic, open surgery should not be seen as risk-free as long as energy sources are used that produce surgical plume and an appropriate capture device should also be used [36].

### 3.4. Clinical Evidences Regarding Laparoscopy in COVID-19 Positive Patients

We encountered 7 case reports published about Covid-19 suspects or infected patients who underwent laparoscopic procedures (appendicectomy, cholecystectomy, perforated ulcer repair, internal hernia repair) [43,44,45,46,47,48,49]. The RT-PCR did not evidence the presence of viral ARN in peritoneal lavage, but, when tested it was present in stools +/− digestive wall in 50% of them. All precautions have been taken and none of the cases resulted in infection of the surgical team. Coccolino et al. [50], on the contrary, communicated that the virus was present in the peritoneal liquid of a patient admitted with a diagnosis of intestinal mechanical obstruction due to small bowel volvulus associated to SARS-CoV-2 pneumonia, treated by open surgery and emphases on the importance of avoiding aerosols generating procedures in such patients (Table 3).

In a case of perforated peptic ulcer and Covid-19 pneumonia, Galvez et al. [45] chose laparoscopic approach, with the pneumoperitoneum evacuated via the laparoscopic high-efficiency particulate air (HEPA) filter, in accordance with the latest recommendations, due to the condition of the patient—obese, under corticotherapy, who could possibly need mechanical ventilation in a prone position. Pawar et al. [46] also reported safe laparoscopic surgery in 12 cases of colorectal cancer using air seal and a HEPA filter during the COVID-19 pandemic. In his opinion, the important advantages of the minimal blood loss, decreased ward stay and minimum intervention by staff for dressing and monitoring are important advantages that should be taken into account and laparoscopy should be used, with all precautions taken, even when there is no possibility of routine testing the patients for SARS-CoV-2.

Mattone et al. [49] reported a case of gangrenous acalculous cholecystitis in a patient hospitalized for COVID-19 pneumonia for 43 days in ICU. He followed the protocol, tempting a less invasive approach, percutaneous drainage, than followed by laparoscopic cholecystectomy. The bile was not positive for SARS-CoV-2, yet the author underlines the ischemic mechanism of gangrenous acalculous cholecystitis, suggesting it might be a consequence of severe acute respiratory distress syndrome of COVID-19 pneumonia which determined vascular insufficiency, responsible of gallbladder wall ischemia. The same founding was noticed by Safari [43].

Although there is no evidence of infection of medical personnel in this way, more data are needed in the investigation of COVID-19 transmission from laparoscopy-related aerosolization.

### 3.5. Regulations for Increasing Safety in Laparoscopic Surgery

Preoperative testing of surgical patients with RT-PCR for SARS-CoV-2 2 is strongly recommended but it does not guarantee lack of infectivity due to a demonstrated false-negative rate of up to 10–30%, including the patients in early incubation period or post-infection, with a minimal viral load at the level of nasopharynx [51]. For this reason, wearing complete PPE, limiting elective hospitalizations, spacing surgeries with keeping 30 min–1 h between them is recommended [51,52,53,54].

Regarding the laparoscopic approach in patients suspected or infected with SARS-CoV-2, the International Endoscopic Surgery Societies have warned since the beginning of the pandemic of the risk of aerosolization of particles, which may have a viral load, given the 19-nCov tropism for the digestive mucosa. If initially these interventions were mostly contraindicated, later, as the medical society learns to coexist with the new virus, the preventive methods necessary to mitigate the infectious risk, were defined more and more coherently. These include appropriate PPE (FFP2 respirator or higher, wrap around goggles or face shield), smoke evacuation devices connected to a filtration system and proper room filtration and ventilation [52,53].

Positive pressure is recommended in the operating room to prevent the penetration of non-sterile air. However, this favors the aerosolization of the particles in the surgical smoke. For this reason, the recommendations during the COVID-19 pandemic are to use negative pressure or if not possible, at least to stop the positive pressure [6,7,8,9,10,11,54,55]. Spacing interventions with a 30 min to 1-h interval, depending on the ventilation possibilities, is also necessary to limit the possibility of contamination.

The surgical smoke filtration systems are proved to be useful in preventing its toxic and infectious potential. SARS-Cov-2 virus size ranges from 0.070–0.075 μm. The recommended filters are HEPA, with an efficiency of 99.97% in removing particles > 0.03 μm diameter or ultra-low particulate air (ULPA) filters, which can filter particles > 0.05 μm size. Although these measures are discussed as safety rules in recent years, taking into account the findings related to the toxic and infectious risk of surgical smoke, their implementation is inhomogeneous, largely lacking in the usual equipment of surgical teams.

All surgical societies (SAGES, EAES, AMASI, IAGES) [6,7,8,9,10,11] have adopted a set of measures to minimize the emission of aerosols during the intervention, consisting in reduced pressure of the pneumoperitoneum, tight incisions to prevent leakage at the trocar orifices, minimum use of energy devices and use of cold hemostasis whenever possible, integrated insufflation devices comprising smoke evacuation and filtration mode, HEPA or ULPA type and valve type valves to prevent gas loss when changing the instrument. Hand-assisted surgery and specimen removal are associated with significant leakage of CO_2_, as a consequence, they must be performed after desufflation. Surgical drains should be utilized only if necessary (Table 4). Any deviation from best practice or mistakes while using these precautions may represent a higher risk of pollution and dangerous exposure for the entire operation room (OR) staff and subsequent personnel in the OR, which represents additional psychological stress for the operating team. For this reason, an additional precaution is to limit the personnel in the OR, to avoid complicated maneuvers and to involve the experienced personnel [52,53,54].

Adapting laparoscopic protocols to the pandemic era, by the use of filtration devices, smoke evacuation devices connected to trocars, the use of self-sealing trocars connected to negative pressure suction may add significant financial burden to a health care system which is already under maximal pressure [40]. Several authors developed different cost-effective filtration systems, both having as the central piece an HME (Heat and Moisture Exchangers) filter, which somehow is connected to a trocar to allow save dessuflation of CO_2_ and surgical smoke [56,57,58,59,60]. HME filters have high resistance to flow and, most important, bacterial and viral filtration efficiency of ≥ 99.999%.

### 3.6. The Impact of the Novel Guidelines upon Surgical Practice

The COVID-19 pandemic markedly disrupted the usual surgical practice. The lack of large-scale testing for all patients admitted for surgical pathologies is still a major challenge for many countries. Limitations in screening capacity, unsatisfactory delays to result reporting (especially in management of acute emergencies) and a high false negative rate (up to 20%), have complicated preoperative screening [61]. The filtration systems for laparoscopic smoke are not a standard for many surgical departments. All these led to partial compliance for laparoscopic safety guidelines, with different consequences upon surgical practice. In a research about acute appendicitis management in UK during COVID pandemic, open surgery cases increased from 0.4% to 56%, with an increase of conservative management in mild cases [62]. In a survey conducted by Lazaridis, vast majority of the participants (88.7%, *n* = 149) would not consider performing open surgery instead of laparoscopy for post- bariatric patients who require emergency surgery during the COVID-19 pandemic, due to the increased post-operatory complications associated to open approach [63].

Similar conclusions are communicated by Manzia et al. [64]. In a survey regarding the preferred approach to gallbladder diseases, only 5.6% of participants chose to perform an open cholecystectomy in all patients and 13.9% of participants would use an open approach in patients with known or suspected SARS-CoV-2 infection, the others answered they would still use the standard laparoscopic approach, due to the fast recovery and low level of complications [64]. In this regard, The Royal College of Surgeons recommends to consider laparoscopy only in selected individual cases, where the clinical benefit to the patient substantially exceeds the risk of potential viral transmission to the surgeons and the theatre teams in that particular situation.

## 4. Discussion

Apart from the observance of these norms or in the technical impossibility to respect them in full, the decision to choose between the classic and the laparoscopic approach remains, according to up to date regulations, at the discretion of the surgical team, if the clinical benefit to the patient substantially exceeds the risk of potential viral transmission in that particular situation. This is an ethical challenge for the surgeons well used to the advantages of laparoscopic surgery—decreased hospital stays, quick recovery, less postoperative complications, including less risk of contracting coronavirus, less consumption of health resources. In many hospitals, routine testing of all surgical patients, although ideal, cannot be performed for financial and logistical reasons. The question is whether there is solid evidence to take the step back to classical surgery, depriving patients of the benefits of the minimally invasive approach just because they might be suspected of SARS-CoV-2 infection?

Studies show that patients infected with SARS-CoV-2 who underwent abdominal surgery have high postoperative morbidity and mortality, so surgery should be avoided or performed in the less invasive method [50]. “Primum non nocere” is a mainstay to most surgeons and something that most surgeons have lived by. There are strong opinions advocate that in particular cases, for instance obese patients with internal hernia or patients with COVID-19 pneumonia, who most probably could require prolonged corticotherapy and intubation, the act of laparotomy is a harm itself [65]. In the opinion of Gupta et al. [40], implementing the additional protective measures provided by current guidelines to limit aerosolization would be sufficient to prevent the theoretical risk of disease in this way. Other authors [36,39,60] show that the current evidence is insufficient to give up the benefits of laparoscopy, however, the key to antiviral protection remains the wearing of appropriate protective equipment along with methods of filtering the artificial pneumoperitoneum.

There is no evidence comparing the COVID-19 safety of open surgery and minimally invasive surgery. Open surgery also carries risks, if aerosolization procedures are used (e.g., use of monopolar energy) and the infectious potential might be even bigger rather than using laparoscopic approach with filtering of laparoscopic smoke. So, we do not really know that if we opt for open surgery we opt for the ‘safer’ option.

## 5. Conclusions

Previously published data showed there is possible infectious and toxic risk related to surgical smoke but not particularly proven for SARS-CoV-2. As the COVID-19 pandemic seems to be still far from ending, healthcare personnel must up-date the clinical protocols, to increase safety but without stepping back from the achievements of modern medicine. The impact of avoiding minimal invasive approach could be a health burden due to prolonged hospitalization and post-operatory complications.

On the other hand, in certain situations, the doctors could be faced with ethical dilemmas, as they have to decide whether to opt for their safety or to choose the less harmful option for their patients. Human resource is the most important in health and must be protected, even the risk is low. Respecting the strict regulations regarding PPE remains the most effective protective measure to mitigate the infectious risk both for open and laparoscopic surgery. Implementing standardized filtration systems for pneumoperitoneum and smoke evacuation during laparoscopy, although increases costs, is necessary to increase the safety of minimally invasive surgery during COVID-19 pandemic and it will probably remain a routine also in the future. Although laparoscopy was firstly regarded with extreme prudence, the effect of COVID-19 pandemic will be most probably a progress in the safety regulations regarding the laparoscopic smoke, drawing the attention upon this overlooked subject. As a professional, one must keep in mind the evidence and take the best decision in specific challenging cases.

## Figures and Tables

**Table 1:** Dimensions of aerosols generated by different energy sources used in surgery.

Energy Source	Dimensions of Aerosols (μm)
Electrocautery	0.007–0.420
Ultrasonic scalpel	0.35–6.5
Laser	0.1–0.8

**Table 2:** Health risks related to the surgical plume in the published literature.

Article	Author	Year	Source	Procedure	Evidences
Preliminary study of electrocautery smoke particles produced in vitro and during laparoscopic procedures [12].	DesCoteaux J.G.	1996	Surgical Endoscopy	Laparoscopic smoke	Smoke from 5 laparoscopic procedures was analyzed; 2 types of particles were identified: Large, irregular particles (2–25 μm): cellular fragments Small homogeneous spheres (0.1–0.5 μm) composed of sodium, magnesium, calcium and potassium salts.
Chemical composition of surgical smoke formed in the abdominal cavity during laparoscopic cholecystectomy-Assessment of the risk to the patient [15].	Dobrogowski, M.	2014	International Journal of Occupational Medicine and Environmental Health	Laparoscopy	Vast array of chemical compounds, including aliphatic and aromatic hydrocarbons such as benzene and its alkyl derivatives, as well as aldehydes, nitriles, amines, polychlorinated dioxins and furans, including the highly toxic 2,3,7,8-TCDD are detected in the surgical smoke
Surgical smoke [17].	Fan, J.K.-M.	2009	Asian Journal of Surgery	Electro-cautery, laparoscopy	Various chemicals (hydrogen cyanide, benzene, hydrocarbons, nitriles, fatty acids and phenols), viruses and bacteria, viable cells were identified in surgical smoke in reviewed studies N95 grade or equivalent respirator offers the best protection against surgical smoke HEPA filters or equiv. are necessary to remove smoke
Health risk to medical personnel of surgical smoke produced during laparoscopic surgery [16].	Dobrogowski, M.	2015	International Journal of Occupational Medicine and Environmental Health	Laparoscopy	Toxic chemicals are present in the surgical smoke It is necessary to remove surgical smoke from the operating room in order to protect medical personnel
Detecting hepatitis B virus in surgical smoke emitted during laparoscopic surgery [26].	Kwak H.D.	2016	Occupational and Environmental Medicine	Laparoscopy	HBV was detected in laparoscopically smoke in 10 of the 11 infected patients The infectious risk for surgeon was not assessed
Surgical smoke and infection control [27].	Alp E.	2006	The Journal of Hospital Infection	Laser. Electrocautery laparoscopy	Bio-aerosols with viable and non-viable cellular material with possible risk of infection (human immunodeficiency virus, hepatitis B virus, human papillomavirus) Irritation to the lungs leading to acute and chronic inflammatory changes cytotoxic, genotoxic and mutagenic effects increased protection needed by
Activated carbon fiber filters could reduce the risk of surgical smoke exposure during laparoscopic surgery: application of volatile organic compounds [29].	Choi S.H.	2018	Surgical Endoscopy	Laparoscopy	Activated carbon filters reduce by 85% the concentration of chemicals in evacuated pneumoperitoneum
Contamination resulting from aerosolized fluid during laparoscopic surgery [23].	Engelhardt R.	2014	Journal of the Society of Laparoscopic & Robotic Surgeons	Laparoscopy	Consistent environmental contamination of blood and body fluid during rapid evacuation of the pneumoperitoneum. Using wrap-around style glasses/shields and protective masks prevent contamination of mucous membranes with viruses like HIV and VHC
Blood and body fluid splashes during surgery—The need for eye protection and masks [24].	Davies C.G.	2007	Annals of the Royal College of Surgeons of England	Laparoscopy	50% risk of contamination with aerosolized blood in laparoscopic surgery Most exposure to projectile blood and body fluid occurs towards the end of a case when ports are removed and pneumoperitoneum is released via the port sites
Composition of volatile organic compounds in diathermy plume as detected by selected ion flow tube mass spectrometry [19].	Moot A.R.	2007	ANZ Journal of Surgery	laparoscopy	1 g tissue burned by electrocautery = same carcinogens as in 6 cigarettes smoked/day Smoke filtration is needed
Awareness of surgical smoke hazards and enhancement of surgical smoke prevention among the gynecologists [28].	Liu Y.	2019	Journal of Cancer	Various laser gynecological procedures	HPV contamination lead to laryngeal papilloma and HPV16 tonsillar squamous cell cancer in 4 reported cases Surgical smoke contains chemicals, blood and tissue particles, bacteria and viruses, which has been shown to exhibit potential risks for surgeons, nurses, anesthesiologists
Microbiologic activity in laser resurfacing plume and debris [21].	Capizzi P.J.	1998	Lasers in Surgery and Medicine	CO_2_ laser resurfacing	Smoke from laser surfacing was analyzed for 13 patients; in 38% of cases coagulase-negative *Staphylococcus* was present; one of these cases associated *Corynebacterium*, one *Neisseria* no viral positive culture
Laryngeal papillomatosis with human papillomavirus DNA contracted by a laser surgeon [30].	Hallmo P.	1991	European Archives of Oto-Rhino-Laryngology	Laser, not specified	Laryngeal papillomatosis secondary to HPV types 6 and 11 in a laser surgeon probably due to transmission via surgical smoke from genitals papilloma
Papillomavirus in the vapor of carbon dioxide laser-treated verrucae [31].	Garden J.M.	1988	JAMA	CO_2_ laser	Vapor produced by the carbon dioxide laser during the vaporization of papillomavirus-infected verrucae showed intact viral DNA content. Two models were used for evaluation: an in vitro cutaneous bovine fibropapilloma and an in vivo human verruca model.
Viral disease trans-mitted by laser-generated plume (aerosol) [32].	Garden J.M.	2002	Archives of Dermatology	CO_2_ laser	Bovine papillomavirus–induced cutaneous fibropapillomas were exposed to the carbon dioxide laser. The laser plume was suctioned reinoculated onto the skin of calves, producing HPV infection
Human immunodeficiency virus-1(HIV-1) in the vapors of surgical power instruments [25].	Johnson G.K.	1991	Journal of Medical Virology	CO_2_ laser	HIV-1 can remain viable in cool aerosols generated by certain surgical power tools
Dissemination of melanoma cells within electrocautery plume [33].	Fletcher J.N.	1999	Americal Journal of Surgery	CO_2_ laser	Pellets of B16-F0 mouse melanoma cells were cauterized and the plume collected into culture medium; viable melanoma cells were collected and grown in culture
Human papillomavirus DNA in surgical smoke during cervical loop electrosurgical excision procedures and its impact on the surgeon [34].	Zhou Q.	2019	Cancer Management and Research	Loop electrosurgical excision procedures (LEEPs)	HPV DNA in surgical smoke produced by LEEP ^1^ The nasal epithelial cells of two surgeons were positive for HPV DNA, the same type as those of resected lesion

^1^ Loop electrosurgical excision procedure.

**Table 3:** Reports of COVID-19 patients who underwent laparoscopic surgery.

Paper	Surgery	Patient’s Status	Sample RT-PCR Test for SARS-CoV-2	Result	Prevention Intraoperatory Measures
Ngaserin S.H. [45]	Laparoscopic appendicectomy	Asymptomatic, COVID-19+	Peritoneal fluid	−	PPE ^1^
Safari S. [43]	1. Laparoscopic cholecystectomy 2. Perforated ileum. Chron disease–laparotomy 3. Open appendectomy 4. Peptic ulcer repair-laparotomy	COVID-19+, symptomatic	Peritoneal fluid Digestive wall Feces Abdominal fat, omentum	− + + −	PPE
Galvez A. [44]	Perforated ulcer repair	COVID-19 pneumonia	Gastric wall	+	PPE Laparoscopic HEPA ^2^ filter
He L. [48]	Perforated ulcer repair	COVID-19 pneumonia	none	−	PPE Negative pressure in OR ^3^ (−5 kPa) Skilled laparoscopic expert to minimize risk
Pawar T. [46]	Laparoscopic Anterior Resection for Rectal Carcinoma	COVID-19 suspects (not tested)	none		PPE Air seal (CONMED, Utica, NY) and high-efficiency particulate air (HEPA) filter was utilized for safe gas evacuation
Lovece A. [47]	Subtotal cholecystectomy	COVID-19 pneumonia	none		PPE Low Pneumoperitoneum pressure (9 mmHg) Low energy power Pneumoperitoneum was evacuated by the suction device before trocar removal and specimen extraction

^1^ Personal protective equipment; ^2^ High-efficiency particulate air; ^3^ Operation Room.

**Table 4:** Recommendations of international societies for safe laparoscopic surgery during COVID-19 pandemic.

Precautions	SAGES/EAES	ELSA	ALSGBI	ESGE
Sizing the incisions	Incision ports as small as possible	Purse-string suture or disposable trocar with skin blocking system should be used	Balloon/self-sealing trocars	**+**
Creating pneumoperitoneum using the most familiar technique		**+**		
Low CO_2_ insufflation pressure (10–12 mmHg)	**+**	**+**	**+**	**+**
Minimal use of electrocautery	**+**	**+**	**+**	
Low power setting of electrocautery	**+**	Prevent plume formation by low energy, keeping instruments clean, limited dissection, frequent suction		
Ultra-filtration of smoke	HEPA/ULPA filters are strongly recommended	Passive or active filtration systems of pneumoperitoneum	**+**	**+**
Disposable instruments to prevent viral contamination		**+**		
Drain only if necessary		**+**		
Attach a CO_2_ filter to one of the ports for smoke evacuation if needed, do not open the tap of any ports unless they are attached to a CO_2_ filter or being used to deliver the gas				**+**
Minimize introduction and removal of instruments through the ports as much as possible				**+**
All pneumoperitoneum safely evacuated via filtration system before closure, trocar removal, specimen extraction or conversion to open	**+**	**+**	**+**	**+**

SAGES: Society of American Gastrointestinal and Endoscopic Surgeons; EAES: European Association for Endoscopic Surgery; ELSA: Endoscopic and Laparoscopic Surgeons of Asia; ALSGBI: Association of Laparoscopic Surgeons of Great Britain and Ireland; ESG: European Society for Gynecological Endoscopy. HEPA: High-efficiency particulate air; ULPA: Ultra Low Particulate Air.
